# User Preference-Based Video Synopsis Using Person Appearance and Motion Descriptions

**DOI:** 10.3390/s23031521

**Published:** 2023-01-30

**Authors:** Rasha Shoitan, Mona M. Moussa, Sawsan Morkos Gharghory, Heba A. Elnemr, Young-Im Cho, Mohamed S. Abdallah

**Affiliations:** 1 Computer and Systems Department, Electronics Research Institute (ERI), Cairo 11843, Egypt; 2 Faculty of Computer and Software Engineering, Misr University for Science and Technology, 6th of October City 12566, Egypt; 3 Department of Computer Engineering, Gachon University, Seongnam 13415, Republic of Korea; 4 Informatics Department, Electronics Research Institute (ERI), Cairo 11843, Egypt

**Keywords:** motion descriptors, visual descriptors, tracklets, whale optimization, video abstraction

## Abstract

During the last decade, surveillance cameras have spread quickly; their spread is predicted to increase rapidly in the following years. Therefore, browsing and analyzing these vast amounts of created surveillance videos effectively is vital in surveillance applications. Recently, a video synopsis approach was proposed to reduce the surveillance video duration by rearranging the objects to present them in a portion of time. However, performing a synopsis for all the persons in the video is not efficacious for crowded videos. Different clustering and user-defined query methods are introduced to generate the video synopsis according to general descriptions such as color, size, class, and motion. This work presents a user-defined query synopsis video based on motion descriptions and specific visual appearance features such as gender, age, carrying something, having a baby buggy, and upper and lower clothing color. The proposed method assists the camera monitor in retrieving people who meet certain appearance constraints and people who enter a predefined area or move in a specific direction to generate the video, including a suspected person with specific features. After retrieving the persons, a whale optimization algorithm is applied to arrange these persons reserving chronological order, reducing collisions, and assuring a short synopsis video. The evaluation of the proposed work for the retrieval process in terms of precision, recall, and F1 score ranges from 83% to 100%, while for the video synopsis process, the synopsis video length compared to the original video is decreased by 68% to 93.2%, and the interacting tube pairs are preserved in the synopsis video by 78.6% to 100%.

## 1. Introduction

Nowadays, video surveillance cameras are used as a crucial device everywhere, inside and outside buildings, to monitor people and prevent law-breaking, violence, kidnapping, etc. However, these cameras produce massive videos with an extensive duration. Thus, searching for a certain activity within these videos involves browsing the entire video content from the beginning to the required activity, which is considered an exhausted and time-consuming operation. Different solutions are proposed to tackle this challenge by summarizing these videos as video fast-forwarding [1], video abstraction [2], video montage, and video summarization [3]. Some of these approaches summarize the video by omitting the inactive frames or selecting the keyframes that lead to losing the original video dynamic relations. However, the other approaches shift many space–time regions in both time and space, and then stitch them together, leading to obvious stitching seams in summary.

**Figure 1:**
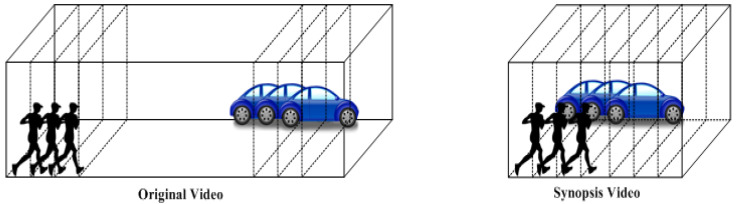
Video synopsis idea.

Recently, the research community presented a new smart technology that can create a condensed representation from the original video without losing significant activities from its content. This smart technology is called video synopsis. This technology improves the functionality of the surveillance videos because it helps the final user lessen the browsing hours of the captured video to minutes or seconds. Moreover, video synopsis affords a video condensation technique that relies on activities rather than those based on frames, so it achieves higher efficiency, as it provides the opportunity for better condensation due to its accurate analysis of video details. The video synopsis is generated by shifting all the objects in time to be presented simultaneously and creating a shorter video with a maximum number of activities, as shown in Figure 1. The video synopsis framework incorporates four principal modules: object detection, object tracking, optimization of the cost function to obtain optimal temporal rearrangement, and, finally, segmenting and stitching the objects’ activities to the generated background.

The video synopsis challenge is to obtain the activities’ best rearrangement to exhibit most of these activities in the shortest time span without collisions of activities. Recently, various video synopsis procedures have been addressed to tackle this challenge. Li et al. [4] introduced a solution for the object collision issue in video synopsis by suggesting scaling down the colliding objects. In this technique, the objects are stirred in temporal domains, then, if a collision is recognized, the objects’ sizes are minimized. A metric is used in an optimization step to represent the minimization factor for each object. Although the problem of object collision has been curtailed technically, the suggested approach might upset the user. Reducing the object size results in showing the video synopsis in an artificial view. A car and a person displayed in a scene close to each other may appear to have equal sizes.

He et al. [5,6] defined collisions’ status between objects activities, namely, collision-free, collision in the same direction, and collision in opposite directions, which result in a step further in the analysis of activity collision. They also proposed a collision graph-based optimization strategy to promote filling and rearrangement of the activity tubes in a deterministic manner to reduce the computational complexity. Hence, a further elaborate activity collision analysis is afforded compared to the other studies of video synopsis. Besides the improvements accomplished via collision minimization, some other metrics are disregarded as a chronological sequence and activity cost. Accordingly, finding the optimal temporal rearrangement of the activities using their optimization approaches still needs to be developed.

Moreover, Nie et al. [7] presented a video synopsis technique, which aims to move the objects in the time and spatial domains to produce a condensed video, as well as to reduce their collision. On the other hand, Lin et al. [8] introduced a distributed-based processing approach to decrease the complexity of computation for creating a video synopsis. The original video is partitioned into several segments. Each segment is assigned to a specific computer to gain the merits of the multi-core capabilities. Raman [9] suggested a video synopsis procedure to maintain the relationships among objects. In this method, the interaction between objects is measured utilizing the differences among various objects’ tubes. If the difference is higher than a predetermined threshold value, the tubes are consolidated to generate a tube set. Ghatak et al. [10] presented an effort for minimizing activity loss, collision number, and cost of temporal consistency. They proposed an improvement for the energy minimization strategy. They utilized a hybrid approach of both the simulated annealing (SA) and the teaching–learning-based optimization (TLBO) algorithms to reach a globally optimal solution besides a reduced computational processing time.

Additionally, Ghatak and Rup [11] evaluated the performance of various optimization techniques for energy minimization for application of video synopsis, namely, SA, cultural algorithm (CA), TLBO, forest optimization algorithm (FOA), gray wolf optimizer (GWO), non-dominated sorting genetic algorithm-II (NSGA-II), JAYA algorithm, elitist-JAYA algorithm, and self-adaptive multi-population-based JAYA algorithm (SAMP-JAYA). This study infers that the present meta-heuristic methods are incapable of reducing energy regularly, though these techniques are popularly applied to minimize the energy for generating video synopsis. In [12], Ghatak et al. suggested improving the energy minimization procedure utilizing a hybridized algorithm combining SA and JAYA. Yao et al. [13] suggested using the genetic algorithm (GA) to produce a new formula for minimizing the energy function. Furthermore, Xu et al. [14] recommended an optimization scheme based on GA to resolve the object tubes merging problem in originating a video synopsis. They deduced that the method based on GA outperforms the one based on SA in terms of information loss and time consumption. Moussa and Shoitan [15] utilized particle swarm optimization to arrange the object tubes using an energy minimization function to decrease the collision, preserve the chronological order, and relate the objects.

Huang et al. [16] confirmed the prominence of the online optimization techniques, which allow the rearrangement of tubes at the detection time without needing to wait for the process of optimization to begin. The most significant issue with their suggested approach is ignoring the activity collision states totally to enhance the operating time performance. Another defect of their suggested optimization technique is using a threshold value that is manually determined instead of using a decision technique. A trade-off issue between the operating time and the ratio of condensation also appears, which results in precision reduction.

Some other studies have addressed the video synopsis from other points of view. Feng et al. [17] introduced a background generation method by choosing video frames with the most activities and background variations in images. Baskurt and Samet [18] stated an adaptive background generation technique to increase the object detection robustness. Afterward, Feng et al. [19] proposed a tracking method to overcome object blinking, which is responsible for the appearance of ghost objects in video synopsis. Baskurt and Samet [20] proposed a tracking approach that concentrates on long-term tracking to realize each target object with only one activity in the created video synopsis. Lu et al. [21] concentrated on the defects of object detection techniques, such as shadow and breaks of object tracking that yield to minimize the content analysis efficiency. Hsia et al. [22] focused on introducing an efficient searching technique for an object activity database to produce a synopsis video. Therefore, a range tree technique was suggested to select object tubes and reduce the algorithm complexity efficiently. Ghatak et al. [23] explored the notion of the multi-frame and scale procedure together with generative adversarial networks (MFS–GANs) to extract the foreground. A hybrid algorithm, including both grey wolf optimizer (GWO) and SA (HGWOSA), is suggested as an optimization algorithm to achieve the globally optimal result with a low computation cost.

On the other hand, different researchers address grouping similar activities in the video synopsis system based on a matching strategy or user-defined query. Lin et al. [24] suggested an approach for video synopsis generation incorporating clustering activities and anomaly detection, object tracking, and optimization. Namitha and Narayanan [25] provide a technique to maintain relationships among object tubes within the input video in the synopsis video. In the first stage, a recursive algorithm for grouping tubes is offered for finding the interaction behavior between tubes and grouping relevant tubes to create tube sets. The second stage aims to optimally rearrange the tubes in the video synopsis system using a spatial–temporal cube voting approach. Finally, an algorithm that relies on measuring the entropy for tube collisions to estimate the synopsis video duration is introduced. Pritch et al. [26] proposed a real-time video synopsis according to a query from a user to show the activities during a specific duration on an endless webcam or surveillance cameras. Pritch et al. [27] introduced a video synopsis showing similar activities with the same appearance and motion features.

Ahmed et al. [28] generated a video synopsis technique for traffic monitoring application using a user query based on object attributes, such as the object classes and movements. First, the moving objects are tracked and classified using deep learning into different categories (e.g., car, pedestrian, and bike). Second, a query is obtained from a user then the tubes fulfilling the query are blended on the background frame for synopsis generation. Namitha et al. [29] proposed an interactive visualization technique to build the synopsis video. Some basic visual features, such as color and size, and some spatial features are used to retrieve certain objects to be addressed in the synopsis. YOLOv3 and Deep-SORT are utilized for the detection and tracking stage. The techniques perform tube grouping to preserve relations between objects and use a space–time cube algorithm to arrange the tube groups in a predefined synopsis length.

Although all the aforementioned clustering and user query-based methods solve the issue of creating an unsatisfactory synopsis video for a crowded scene due to the collision, they do not consider specific appearance attributes such as gender, age, carrying something, having a baby buggy, and upper and lower clothing color. These attributes can help the camera monitor find a suspected person using an appearance description or a particular action happening in the scene. Thus, to achieve this goal, an analysis must be accomplished on the recorded video, depending on the user’s requests; a synopsis video will be constructed to attain the requirements. Accordingly, the process involves video analysis to retrieve the user appeal and an optimization stage to build the synopsis efficiently.

In this paper, a framework that sustains a smart-condensed video synopsis system relying on prescribed user recommendations is developed. The proposed system utilizes a highly detailed user-defined description for the desired persons, and then arranges them using an intelligent-optimization technique, the whale optimization algorithm [30], to construct a low-collision condensed synopsis.

The contribution of this work can be abstracted as follows:

- The proposed technique permits the user to stipulate a detailed description of the desired persons in three distinct aspects, precise visual appearance, motion description, and accessing regions of interest in the scene, contrary to the traditional user-query synopsis methods.
- Several detailed distinct descriptions of a person are employed to design a user-defined query. These descriptions incorporate an elaborated person’s visual appearance, motion style, and motion type, and personal behavior concerning the region of interest.
- Persons’ tubes are generated and assembled based on the relationships defined by the user’s query. Furthermore, using an intelligent optimization method, the whale optimization algorithm, the provoked tubes are arranged to construct a highly visually intelligible synopsis video preserving false overlapping between the persons, as well as conserving the correlation time order.

The sections henceforth are arranged as follows: Section 2 describes the details of the proposed approach, Section 3 demonstrates the experimental results, and, finally, Section 4 contains the conclusion.

## 2. Methodology

**Figure 2:**
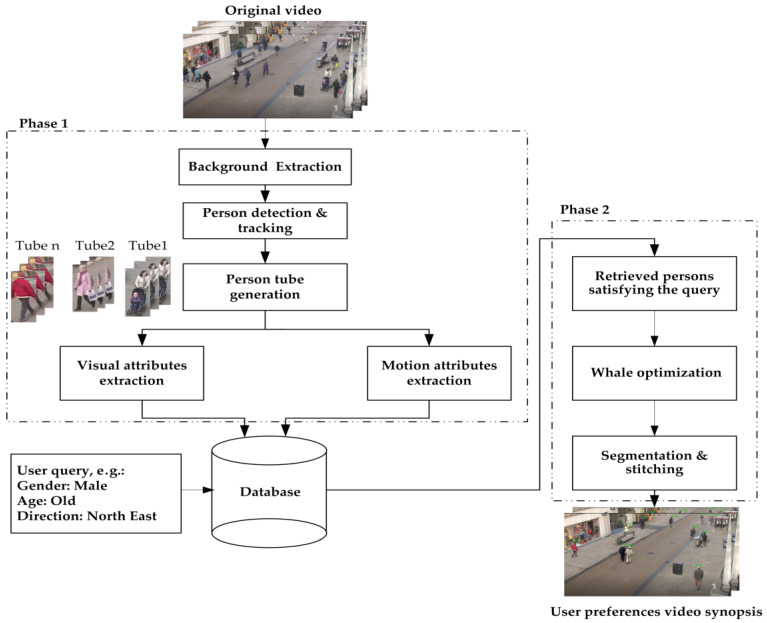
Proposed system architecture.

Although video synopsis is an emerging technology in video analysis research, it faces different challenges, such as creating a synopsis video that involves a suspected person having a specific appearance description consistent with user preferences. In the proposed system, the user submits a query that specifies the detailed descriptions of retrieved desired persons. The descriptions enclose appearance features, such as gender, age (5 age ranges), carrying something or not, having a baby buggy or not, upper clothing color (11 colors), and lower clothing color (11 colors). Furthermore, the user can request to retrieve persons based on a moving direction (8 directions). Moreover, users may desire to retrieve persons entering or exiting a specific region of interest or based on their motion speed. Figure 2 illustrates the proposed system architecture. The suggested system proceeds in two phases, each incorporating several steps. The first phase comprises extracting the background, tracking the existing persons, and generating their corresponding tubes. Moreover, during this phase, visual appearance and motion features are extracted. In the second phase, on the other hand, a user-defined query is used to retrieve the desired person’s tubes. These tubes are then arranged and utilized to construct the synopsis video.

As can be noticed in Figure 2, the first phase commences with the camera monitor selecting the video from the video store that has the required person. Then, the temporal median method is applied to estimate the background. Afterward, the people bounding boxes from the selected video are extracted using the proposed detection and tracking algorithms. Finally, the visual features, comprising gender, age, carrying something or not, having a baby buggy or not, upper clothing color, and lower clothing color, as well as the motion features, according to the motion speed, motion direction, and the person accessing a region of interest, are extracted for each person tube using a person attribute recognition algorithm. On the other hand, in the second phase, the extracted visual and motion features are stored in the database, and according to the user query, the person tubes that satisfy the query are retrieved. Subsequently, a whale optimization algorithm is applied to determine the best starting time of each retrieved person tube that minimizes the synopsis length. Eventually, the retrieved person tubes are segmented and stitched on the estimated background to generate the video synopsis.

### 2.1. Phase 1: Tube Generation and Feature Extraction

This phase aims to detect and track multiple persons to generate tubes corresponding to each person and extract elaborated features for each person’s tube.

#### 2.1.1. Background Estimation

The first step in video synopsis is extracting the background for stitching the generated person tubes. The temporal median method is used to extract the background by applying it to a group of 25 neighboring frames, exploiting the fact that the surveillance videos have fixed backgrounds with little change in the illumination. The background estimation step impacts the visual quality of the synopsis video, but it does not affect the effectiveness of its compression.

#### 2.1.2. Person Tracking and Tube Creation

In this step, the Bytetrack algorithm is utilized to build a motion tube for each person (tracklet), which is a group of bounding boxes throughout the video. The Bytetrack algorithm is carried out in three stages: object detection, object localization, and association. The object detection step is responsible for recognizing objects within the frame. The YOLOX model was adopted for conducting this task. Afterward, the Kalman filter is applied to perform object localization to predict the location of each object in the next frame. The BYTE algorithm is then utilized for the association process to decide whether objects in various frames are related to the same identity. BYTE considers all detected boxes, not only the high scored ones. First, it links high score detected boxes with existing tracklets. Nevertheless, due to occlusion, size variation, and motion blurring, some tracklets are unmatched to a high score detected box. Accordingly, these tracklets are matched to the low detected score boxes. This strategy guarantees a higher tracking performance and less identity switching than traditional multi-object tracking algorithms [31,32].

#### 2.1.3. Visual Appearance Features Extraction

For each tracked person, different attributes describing their visual appearance, such as age, gender, upper and lower clothing colors, etc., are extracted. In the proposed algorithm, one of the part-based person attribute recognition algorithms, the attribute localization module [33] (ALM), is used. The advantage of ALM is that it concentrates on the person parts, improving the person attribute recognition. In the ALM algorithm, each person bounding box is fed into the main network with feature pyramid architecture, then the generated features from different levels are sent to a group of attribute localization modules to apply attribute localization and region-based feature learning for obtaining the attribute vector. Each attribute localization module is designed to serve one attribute at a single level. The features of each pedestrian bounding box are extracted based on the batch normalization inception (BN-inception) network as a backbone network, and each attribute localization module is designed to depend on a simplified spatial transformer network (STN) [34]. The algorithm is trained on one of the person attribute recognition datasets, which is PETA [35].

#### 2.1.4. Motion Features Extraction

Retrieving persons based on their motion in the video is critical in determining who enters various locations regarding the surveillance scope. Furthermore, specifying the motion style affords awareness about the actions taking place in the scene. The proposed system provides some motion information that can assist the individual monitoring the camera to reveal suspected actions. The variations in the location of each person’s bounding boxes are employed to express their movement through three aspects:

Motion style: the speed of change of the bounding boxes’ centroids can state if the person is running, walking, or stopping at a specific area for a while.

Motion direction: the system can determine the route of each person through the 8 main directions (north, south, east, west, north-east, north-west, south-east, south-west);

Accessing regions: the camera monitoring man can specify some regions of interest in the surveillance scope to recognize the persons who entered and exited from these regions.

### 2.2. Phase 2: Persons Retrieval and Synopsis Generation

This phase commences by applying a user-defined query specifying the desired person’s description. The query is constructed by giving the camera monitor a set of options to select the required person’s specifications, such as age, gender, carrying something or not, having a baby buggy or not, lower and upper clothing color, motion style, motion directions, and accessing a region of interest. Then, these attributes from the user query are matched with the extracted attributes for each person tube to select only the matched one. Afterward, the matched persons are segmented and stitched on the estimated background in an optimized order.

#### 2.2.1. Optimization

The visual appearance and motion features extracted from each tube are compared with the user query to determine the matching ones. These person tubes are arranged to preserve their chronological order. The whale optimization algorithm is suggested to organize the appearance order and the person’s starting time depending on a fitness function.

(a) Fitness function

The fitness function guarantees some constraints confrontation, building a synopsis as short as possible, preventing collisions between persons, preserving true collisions and correlation order. Each of these parameters has a weight value that the user can use to fine-tune the degree of importance of each of them. The proposed fitness function is expressed as

(1) $$E\left(t\right)={\kappa E}_{\mathrm{Length}}\left(t\right)+{\alpha E}_{\mathrm{collision}}\left(t\right)+{\omega E}_{{\mathrm{true}}_{\mathrm{colision}}}\left(t\right)+{\gamma E}_{\mathrm{temporal}}\left(t\right)$$

where E_Length_, E_collision_, E_true_collision_, and E_temporal_ represent the synopsis length cost, the activity collision cost, the true collision cost, and the temporal consistency cost, respectively. Moreover, κ,α,ω,γ symbolize synopsis length weight, collision weight, true collision weight, and temporal consistency weight, respectively. E_Length_ is responsible for decreasing the synopsis length as much as possible to not go above the longest tube, while E_collision_ reduces the object’s collision after mapping the object’s tubes. On top of that, the role of E_true_collision_ is to maintain the intersection relation in the original video to be mapped for the synopsis video and E_temporal_ preserves the chronological order of the object’s tubes in the generated synopsis. The whale optimization algorithm aims to minimize this fitness function for finding each tube’s starting time in the synopsis video that reduces the object’s tube collision, decreases the synopsis length, maintains the intersection relations as much as possible, and preserves chronological order. Additionally, the proposed algorithm attempts to maintain the counterpart relation in which the two tubes appear temporally and spatially near each other most of the time. If their temporal relationship exceeds 75% and their spatial distance does not exceed twice the person’s width, both tubes are coupled together. Eventually, the synopsis video is created by stitching the motion tubes of the persons in the order given by the optimization step.

(b) Whale optimization algorithm

The whale optimization algorithm is a heuristic optimization algorithm that mimics the humpback whale’s hunting behavior. Their foraging attitude is named the feeding method through bubble-net, which is performed by creating distinguishing bubbles that take a circle-shaped or ‘9’-shaped path. The simulation of hunting behavior to chase the prey is undertaken by using a spiral model to imitate the mechanism of bubble-net attacking of humpback whales. The mathematical model for the whale optimization algorithm includes a model for encircling prey, the spiral bubble-net attacking method (exploitation phase), and prey searching (exploration phase).

- Encircling prey

Humpback whales first recognize the prey’s location and then make a circle around it. The whale optimization algorithm assumes that the best current solution is the location of the target prey or close to it. After determining the best search agent, the other search agents will update their positions toward the best search agent. This manner is represented by the following equations:

(2) $$\vec{D}=\left|C\;\vec{X^{*}}\left(t\right)-\vec{X}\left(t\right)\right|$$

(3) $$\vec{X}\left(t+1\right)=\vec{X^{*}}\left(t\right)-\vec{A}\cdot \vec{D}$$

where $\vec{X}$ is a position vector to the best solution so far and is updated at each iteration in case a better solution is found, *t* is the current iteration, and $\vec{A}$ and $\vec{C}$ are coefficient vectors given by the following equations:

(4) $$\vec{A}=2\vec{a}\cdot \vec{r}-\vec{a}$$

(5) $$\vec{C}=2\;\vec{r}$$

where the component $\vec{a}$ is decreased from 2 to 0 linearly through a pre-defined number of iterations and $\vec{r}$ is a random vector that takes the values [0, 1].

- Bubble-net strategy for attacking method (exploitation phase)

In this section, two approaches for designing the mathematical model for the behavior of the bubble-net of the humpback whale are presented:

(a) Shrinking encircling mechanism

The behavior of the bubble-net attacking method is conducted here by decreasing the value of vector $\vec{a}$ and, consequently, vector $\vec{A}$ also decreases and takes a random value in the interval from [−a, a]. The position of the search agent towards the position of the best current agent can be achieved in 2D space by 0≤A≤1.

(b) Spiral updating behavior

First, the distance between the location of the whale (*X*, *Y*) and the location of prey ($X^{*},\;Y^{*}$) is calculated. After that, the spiral equation is constructed between the whale position and the prey position to simulate the helix-shaped movement of the humpback whales, which is described as follows:

(6) $$\vec{X}\left(t+1\right)=\vec{\overset{´}{D}}\cdot \;e^{bl}\cdot \cos\left(2\pi l\right)+\vec{X^{*}}\left(t\right)$$

where $\vec{\overset{´}{D}}=\left|\;\vec{X^{*}}\left(t\right)-\vec{X}\left(t\right)\right|$ is the distance between the location of *i*th whale to the prey location (best solution found so far), *l* is a number taken randomly from the interval [−1, 1], and *b* is a constant value used to define the logarithmic spiral shape.

The humpback whales swim simultaneously around the prey inside a shrinking circle and along a spiral-shaped path. For updating the whales’ positions during the optimization process, there is an assumption that there is a probability value of 50% to choose between using either the shrinking encircling or the spiral path. The mathematical model for the two behaviors is described as follows:

(7) $$\vec{X}\left(t+1\right)\{\begin{array}{c} \vec{X^{*}}\left(t\right)-\vec{A}\cdot \vec{D}\;\;\;\;\;\;\;\;\;\;\;\;\;\;\;\;\;\;\;\;\;\;\;\;\;\;\;\;if\;p<0.5\\ \vec{\overset{´}{D}}\cdot \;e^{bl}\cdot \cos\left(2\pi l\right)+\vec{X^{*}}\left(t\right)\;\;\;\;\;\;\;\;if\;p\geq 0.5 \end{array}$$

- Prey search (exploration phase)

Humpback whales randomly search for prey according to their locations. Consequently, the random values of vector $\vec{A}$ are changed to fluctuate between 1 and −1 to direct the search agent to move far away from a reference whale. In the contraindication to the exploitation phase, the search agent’s position is updated in the exploration phase according to an agent chosen randomly, rather than choosing the best agent found so far, in which the value of $\left|\vec{A}\right|\geq 1$.

(8) $$\vec{D}=\left|C\cdot {\vec{X}}_{rand}-\vec{X}\right|$$

(9) $$\vec{X}\left(t+1\right)={\vec{X}}_{rand}-\vec{A}\cdot \vec{D}$$

In the proposed system, the whale optimization technique is used to optimize the fitness function in Equation (1) to find the best location of the person tube that minimizes the synopsis length. First, the prey’s location is represented by a vector, and its length is equal to the number of person tubes matching the query. Then, each element of this vector is initialized by the starting frame to stitch this person’s tube in the synopsis video. At each iteration, the whale optimization algorithm attempts to find the best starting frame for each person tube that minimizes the optimization function.

#### 2.2.2. Segmentation, Stitching, and Synopsis Creation

The Poisson technique is used to stitch the tubes into the estimated background. However, a mask is first constructed for each object in the bounding box based on specific morphological procedures to obtain the objects without the surrounding background, making the image appear more natural once the object’s tubes have been stitched. Subsequently, the segmented objects are stitched with the estimated background using seamless cloning based on the Poisson method to stitch these extracted objects with the generated background.

## 3. Experiments and Results

This section presents the datasets details, the evaluation metrics and the simulation results of the proposed system on the selected datasets. All the experiments are conducted on an 11th Gen Intel(R) Core (TM) i7-11800H with RAM (32 GB) NVIDIA GeForce RTX 3060 and implemented in Python 3.7 with Cuda toolkit version 11.3.

### 3.1. Dataset

Two datasets were selected to evaluate the proposed system which are Oxford Town Center dataset [36] and Multi-Camera Object Tracking (MCT) dataset [29]. The Oxford Town Center dataset is a CCTV video of pedestrians in a busy street taken from a security camera at the intersection of Cornmarket and Market St. in Oxford, England. The video was recorded at 25 fps with a resolution of 1920 × 1080 and has 7502 frames. The MCT dataset is made up of four sub-datasets, each with three to five cameras with a 320 × 240 resolution. The dataset consists of cameras that were placed in both indoor and outdoor settings, with notable illumination variation between various cameras. The environment is the same across sets 1 and 2. Sets 3 and 4 were recorded at an office building and a parking lot, respectively. In the proposed research, cam1 and cam2 videos from set 1 were used for the evaluation process. The duration of each video is 20 min, recorded at 20 fps with 234 persons, and has 24,000 frames.

**Figure 3:**
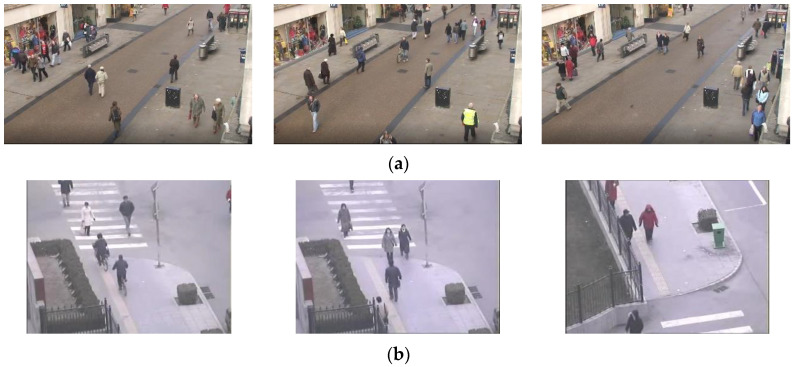
Sample of original frames from the datasets (**a**) Oxford Town Center and (**b**) MCT.

Figure 3 shows sample frames from Oxford Town Center and Multi-Camera Object Tracking (MCT) datasets, respectively.

### 3.2. Evaluation Metrics

The proposed system is evaluated in two stages. The former evaluates the person retrieval process for a user-defined query to examine whether the retrieval system retrieves all the appropriate persons for a specific query or there are missed persons. The latter evaluates the video synopsis performance after stitching all the retrieved persons according to this user-defined query.

The person retrieval process relative to a specific query is assessed using recall, precision, and F1 score. Recall indicates the ratio of correctly retrieved positive objects to all objects in an actual class, precision indicates the ratio of correctly retrieved positive objects to the total retrieved positive objects, and F1 score is a weighted average relation between recall and precision, which can be calculated using the following formulas:

(10) $$\mathrm{Recall}=\frac{\mathrm{TP}}{\mathrm{TP}+\mathrm{FN}}$$

(11) $$\mathrm{Precision}=\frac{\mathrm{TP}}{\mathrm{TP}+\mathrm{FP}}$$

(12) $$F1=2\times \frac{\mathrm{Precision}\times \mathrm{Recall}}{\mathrm{Precision}+\mathrm{Recall}}$$

where TP is the number of true positives, FP is the number of false positives, and FN is the number of false negatives.

For evaluating the video synopsis performance, it was found that no unified standards are defined to measure the accuracy of the synopsis results. However, the earlier methods utilized some metrics to measure the frame condensation ratio (FR), overlap ratio (OR), non-preserved interactions (NPI), and temporal disorder TD [37]. FR represents the ratio of reduction in the resultant synopsis; the lower FR value indicates high condensation. OR determines the false overlap between two different tubes (T_i_ and T_j_) in the synopsis; the overlap is calculated as the average of falsely overlapped pixels all over the synopsis. NPI represents how much the original interacting tube pairs are lost or not retained in the synopsis video. The lower NPI value indicates high interaction preservation. TD measures the ratio of tubes that lost their timing order with respect to the other tubes. FR, OR, NPI, and TD can be defined as

(13) $$\mathrm{FR}=\frac{\mathrm{Number}\;\mathrm{of}\;\mathrm{frames}\in \mathrm{synopsis}}{\mathrm{Number}\;\mathrm{of}\;\mathrm{frames}\in \mathrm{original}\;\mathrm{video}}$$

(14) $$=\frac{\mathrm{Number}\;\mathrm{of}\;\mathrm{overlapped}\;\mathrm{pixels}}{\mathrm{Synopsislength}\times \mathrm{Width}\times \mathrm{Height}}$$

(15) $$\mathrm{NPI}=\frac{\mathrm{Number}\;\mathrm{of}\;\mathrm{interacting}\;\mathrm{tube}\;\mathrm{pair}\;\mathrm{violated}\in \mathrm{synopsis}}{\mathrm{Number}\;\mathrm{of}\;\mathrm{ineracting}\;\mathrm{tube}\;\mathrm{pairs}\in \mathrm{original}\;\mathrm{video}}$$

(16) $$\mathrm{TD}=\frac{1-\sum \left|\mathrm{order}\left(T_{\mathrm{orig}}\right)-\mathrm{order}\left(T_{\mathrm{syn}}\right)\right|}{\mathrm{Number}\;\mathrm{of}\;\mathrm{tubes}}$$

where order(T_orig_) and order(T_syn_) are the time order of a tube in the original and the synopsis video, respectively.

### 3.3. Simulation Results

As mentioned previously, the proposed system is evaluated in two steps: one for the retrieval process and the other for the synopsis. However, there are no rigid experiments to test the proposed system, thus different queries are proposed for retrieval and synopsis performance evaluation. Table 1 presents eight suggested queries for evaluating the proposed system. The queries are selected to be diverse to cover different test cases.

Because each tube contains many bounding boxes for the same person, some of these bounding boxes are unclear due to the occlusion of the person during their movement through the video. The lack of clarity of the bounding box, as a consequence of the occlusion, affects attribute recognition and the retrieval process. As a result, the attributes are recognized for a specific number of bounding boxes of the same person distributed on their presence duration for estimating the person’s attributes correctly and reducing the time required to calculate the attributes for all the bounding boxes.

The retrieval process assessment is attained by calculating recall, precision, and F1 score, which reflects how relevant the results are to the query. Table 2 presents the recall, precision, and F1 score percentages. The table also declares the number of relevant persons in each query, as well as the number of TP, FP, and FN cases. The results represent the average performance of the videos of the two datasets as measured across various queries.

**Figure 4:**
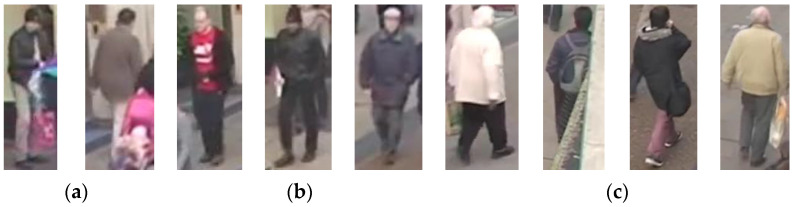
Examples of false-positive retrieval: (**a**) Query2, (**b**) Query4, (**c**) Query8.

**Figure 5:**
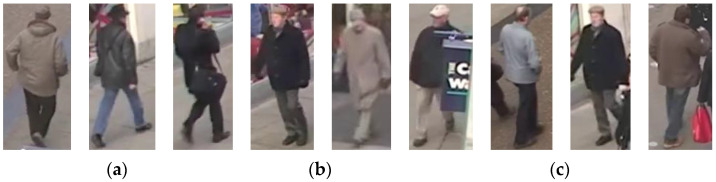
Examples of false-negative retrieval: (**a**) Query1, (**b**) Query7, (**c**) Query8.

It can be noticed from the table that the precision and F1 score values for six out of eight queries are approximately larger than 90%, which reflects that the retrieval system performs well, even though the quality of the surveillance videos is lower. Meanwhile, the suggested system managed to detect all the specified persons in Query4 and missed 14 persons in Query1, although both Query1 and Query4 are related to the gender of the person. This implies that females are detected correctly, while, in some cases, males are incorrectly detected as females. This can happen because of males covering their heads or having colored hair. This result reflects the low recall value of Query1 over Query4. Moreover, Query2 achieved the lowest precision value (83%) because two persons walking beside a baby buggy are recognized as persons having a baby buggy. Thus, the two persons are considered FP. For Query3, the proposed system retrieves the persons wearing the upper clothing with red color. The retrieval system retrieves 31 out of 32 persons correctly; however, the system fails to identify one person’s upper clothing color, which reduces the recall’s value. Furthermore, for Query5, the proposed system distinguishes all persons exiting from a certain region of interest (ROI1). Therefore, recall precision and F1 results are equal to 1. In Query6, only one person is not recognized truly, and they are counted as FN; accordingly, the recall value is reduced. The proposed technique in Query7 searches for old men, and the search results retrieve 43 out of 46 true, and the system fails to recognize three persons as older men, but rather as middle-aged men, so they are considered FN. Additionally, the proposed system identifies a middle-aged person as an older man; thus, it is counted as FP. Therefore, the values of both recall and precision are decreased. On the other hand, the task of Query8 is to retrieve the persons who do not have a bag. The proposed system retrieves 23 false-negative persons due to them walking beside a person holding a bag or due to occlusion, while the system retrieves 29 false-positive persons due to occlusion and having bags with the same color of clothing or background. Figure 4 and Figure 5 demonstrate some false-positive and false-negative cases, respectively.

**Figure 6:**
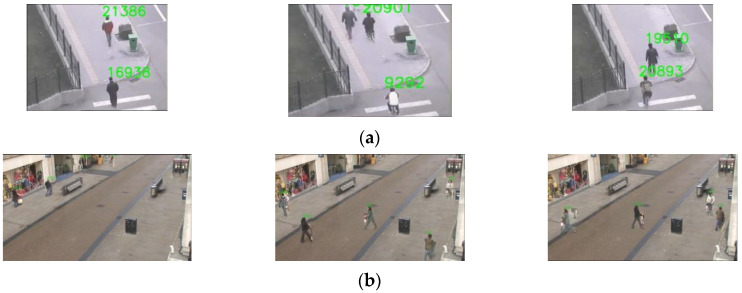

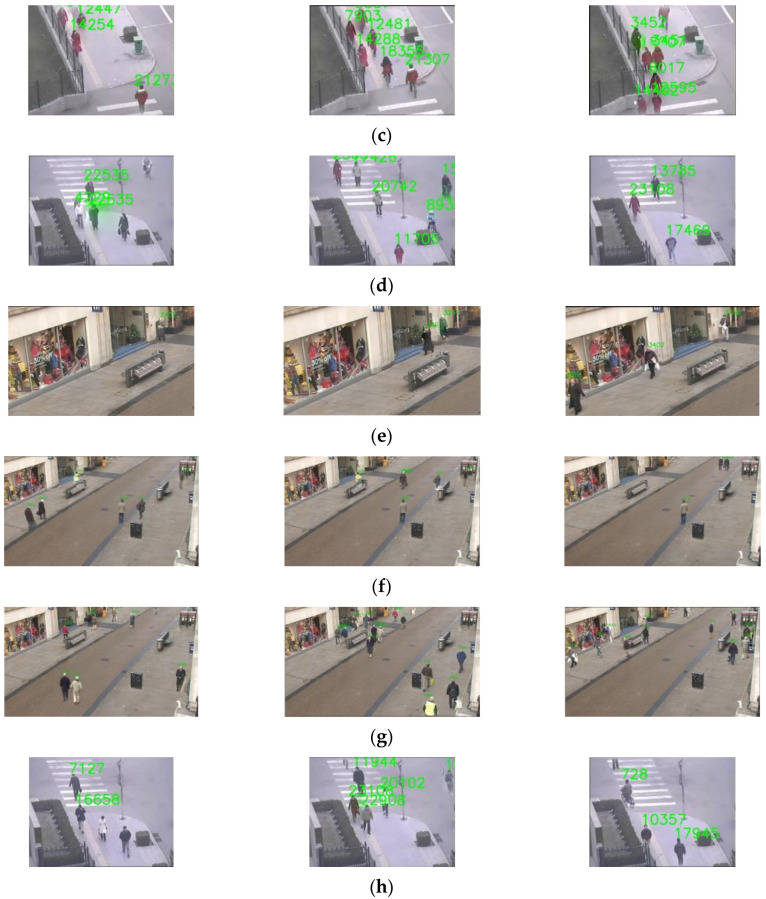
Sample frames of the generated synopsis video from each query: (**a**) Query1, (**b**) Query2, (**c**) Query3, (**d**) Query4, (**e**) Query5, (**f**) Query6, (**g**) Query7, (**h**) Query8.

For evaluating the video synopsis performance, Table 3 presents the different metrics to assess the created synopsis. The whale parameters that are empirically determined to generate the synopsis video are initialized as solutions numbers = 50, the logarithmic spiral shape “b” = 0.5, and the search speed parameter value, “a”, decreases from 2 to 0 through the generations. It can be observed from the results in the table that the synopsis video length compared to the original video is decreased by 68% to 93.2%. The variation in the reduction rate depends on the number of persons needed to be stitched in the video synopsis with less collision and a high time correlation between tubes. Additionally, OR values reflect how the proposed algorithm generates the synopsis video with low collision between the persons. Furthermore, it can be perceived that interacting tube pairs are preserved in the synopsis video by 78.6 to 100%. Some interacting tube pairs are not preserved due to the large number of persons needed to be stitched in the synopsis. It can also be remarked that the proposed algorithm reserves the timing order of the tubes in the synopsis video according to the TD values. TD values are approximately very small for queries with a small number of people and increase slightly for queries that return a large number of people. Figure 6 presents some frames of the generated synopsis videos for each query. As an example, Figure 6a shows frame samples from the generated video synopsis by applying Query1, “Males moving in the opposite direction to the camera,” for the Oxford Town Center dataset, while Figure 6b indicates the frames from the synopsis video by applying Query2, “Persons having a baby buggy” on the MCT dataset. The rest of the queries are described in Table 1. As can be seen in the figure, each query is efficiently satisfied, and the synopsis video stitched the relevant persons in a well-organized manner to ensure a pleasing visual appearance.

Most user-defined query video synopsis methods create their queries according to their applications and needs. Furthermore, a performance comparison cannot be made based only on the measurements of each research since there is no common baseline for widely used datasets, and most of the surveillance videos are not publicly available. Therefore, the proposed and conventional methods are evaluated regarding the types of the user-defined queries and how each method considers collisions and relations between objects in generating a synopsis.

Table 4 demonstrates the evaluation of the proposed method and the conventional methods in terms of the aspects that each technique covers. It can be noticed that one of these methods concentrates on arranging objects with a collision-free and preserving temporal order when creating a synopsis for a period of interest [26].

The other generates the synopsis relative to the object trajectory within a period of interest, attempting to preserve the collision without needing object arrangement [28]. The last method uses a more detailed user-defined query considering the visual, spatial, and temporal features for synopsis generation by arranging the objects to reduce the collision and preserve the tube interactions [29]. Although this method focuses on visual appearance compared to the other methods, their general description attributes cannot help to find suspected persons.

The proposed technique considers three important aspects: retrieving the objects of interest that the user described in detail, keeping the relations between objects, and building a compact synopsis. As can be noticed, the proposed technique covers the various advantages of the previously addressed methods.

Furthermore, Table 5 displays the F1 score, NPI, and TD results of the queries applied in the proposed work, as well as the conventional methods as stated in their research, considering the visual, spatial, and temporal attributes. It can be observed that some algorithm results are missed because the results were obtained from their original papers.

## 4. Conclusions

Building a synopsis video system congruent to users’ demand has yet not been conducted for a large scale of personalized features’ retrieval. In this paper, a video synopsis based on persons’ appearance and motion description features for user request fulfillment is suggested. YOLOX and Bytetrack are presented for the detection and tracking of persons. Afterward, tubes related to a user request are extracted and grouped according to the demanded features. These tubes are arranged based on the whale optimization algorithm to generate a short synopsis video which has fewer collisions between persons, as well as retains true collisions and preserves correlation order. Concerning the retrieval process evaluation, the results demonstrate that there are less false-positive cases (false detected) compared to false-negative cases (missed), which results in high precision values with most queries, thus contributing to the retrieval of as many positive cases as possible, where the precision value ranges from 83% to 100%. On the other hand, false positives and false negatives are affected adversely in some cases due to the similarity in persons’ appearance (as in persons’ gender retrieval cases) or due to occlusion or background similarity. In metrics of synopsis process evaluation, the suggested synopsis system proved its ability in achieving low values of collision and time correlation with all queries. Moreover, it confirms the realization of a high percentage of intersection preservation and time correlation.

## Figures and Tables

**Table 1:** Suggested queries for evaluating the proposed system performance.

Query	Description
Query1	Males moving in the opposite direction to the camera
Query2	Persons having a baby buggy
Query3	Persons wearing red-colored upper clothes
Query4	Females moving towards the camera
Query5	Persons exiting from a certain region of interest (ROI1)
Query6	Persons who stand for some time in a place
Query7	Old men
Query8	Persons who do not have bags

**Table 2:** Retrieval process performance evaluation.

	No. of Relevant Persons	TP	FN	FP	Recall	Precision	F1 Score
Query1	98	84	14	0	0.86	1	0.92
Query2	12	10	2	2	0.83	0.83	0.83
Query3	32	31	1	0	0.97	1	0.99
Query4	266	266	0	20	1	0.93	0.95
Query5	3	3	0	0	1	1	1
Query6	7	6	1	0	0.86	1	0.92
Query7	46	43	3	1	0.93	0.98	0.96
Query8	234	211	23	29	0.9	0.88	0.89

**Table 3:** Video synopsis performance evaluation.

	No. of Persons in Synopsis	No. of Frames in Synopsis	Average FR	Average OR	Average NPI	Average TD
Query1	84	4198	0.125	0.012	0.125	0.082
Query2	12	1320	0.176	0.001	0	0.04
Query3	31	2380	0.078	0.017	0	0.035
Query4	286	12,710	0.246	0.01	0.06	0.259
Query5	3	509	0.068	0	0	0
Query6	6	2397	0.32	0.0003	0	0.008
Query7	44	2046	0.273	0.009	0.056	0.138
Query8	240	13,332	0.271	0.008	0.214	0.243

**Table 4:** Evaluation of the proposed and conventional methods.

	Objects Arrangement	Considered Object Relations	Visual Query	Spatial Query	Temporal Query
Pritch [26]	Yes	- Prevent collision - Preserve temporal order	---	---	- Period of interest
Ahmed [28]	No	- Prevent collision	---	- Object trajectory	- Period of interest
Namitha [29]	Yes	- Prevent collision - Keep interactions	- Color - Type - Size	- Moving in one of the eight main directions - Object path	- Motion speed
**Proposed**	Yes	- Prevent collision - Keep interactions - Preserve temporal order	- Gender - Age - Carrying something or not - Having a baby buggy or not - Upper clothing color - Lower clothing color	- Moving in one of the eight main directions - Entering a region. - Exiting from a region	- Motion speed - Waiting at a region

**Table 5:** Results of the proposed algorithm and the conventional methods in terms of F1, NPI and TD.

	Method	F1	NPI	TD
Visual/color	Pritch [26]	-	0.67	1.562
	Li X [4]	-	1	0.189
	Ahmed [28]	-	0.31	1.491
	Moussa [15]	-	0.54	1.422
	Namitha [29]	0.85	0	1.131
	**Proposed**	0.99	0	0.035
Spatial/IoU	Pritch [26]	-	0.45	7.534
	Li X [4]	-	1	2.929
	Ahmed [28]	-	0.4	7.402
	Moussa [15]	-	0.56	6.561
	Namitha [29]	0.96	0	6.732
	**Proposed**	1	0	0
Temporal/speed	Pritch [26]	-	0.28	1.732
	Li X [4]	-	1	0.566
	Ahmed [28]	-	0.24	1.467
	Moussa [15]	-	0.31	1.582
	Namitha [29]	0.75	0	1.381
	**Proposed**	0.92	0	0.008
